# Safety, tolerability, pharmacokinetics, and antitumour activity of oleclumab in Japanese patients with advanced solid malignancies: a phase I, open-label study

**DOI:** 10.1007/s10147-022-02242-5

**Published:** 2022-11-07

**Authors:** Shunsuke Kondo, Satoru Iwasa, Takafumi Koyama, Tomoko Fujita, Ko Sugibayashi, Kosho Murayama, Noboru Yamamoto

**Affiliations:** 1 National Cancer Center Hospital, 5-1-1, Tsukiji, Chuo-ku, Tokyo, 104-0045 Japan; 2 AstraZeneca K.K., 3-1, Ofuka-cho, Kita-ku, Osaka, 530-0011 Japan; 3 AstraZeneca K.K., 3-1-1, Shibaura, Minato-ku, Tokyo, 108-0023 Japan; 4 Present Address: AstraZeneca, 136 Hills Road, Cambridge, CB2 8PA UK

**Keywords:** Advanced solid malignancies, Antitumour activity, Oleclumab, Pharmacokinetics, Phase I, Safety

## Abstract

**Background:**

Cluster of differentiation (CD) 73-targeted immunotherapy and CD73 inhibition may reduce adenosine production, which can augment the host and/or immunotherapy response to tumours. We aimed to assess the safety and tolerability, pharmacokinetics, and antitumour activity of oleclumab, an anti-CD73 monoclonal antibody, in adult Japanese patients with advanced solid malignancies resistant to standard therapy.

**Methods:**

In this phase I, single-centre, open-label study, patients received oleclumab 1500 mg (Cohort 1) or 3000 mg (Cohort 2) intravenously every 2 weeks.

**Results:**

In total, six patients were enrolled in the study (three in each cohort), and all six patients received the study treatment. The median patient age was 56.0 years and 4/6 were males. All patients (100%) reported adverse events (AEs) during the study; five (83.3%) patients reported AEs related to the study treatment. One (16.7%) patient reported a Grade 3 AE (neutrophil count decreased) that was not related to the study treatment. No AEs with an outcome of death were reported, and no patients reported AEs or serious AEs leading to oleclumab discontinuation/dose interruption. No dose-limiting toxicities were reported, and no patient discontinued due to an AE related to the study treatment. Oleclumab exposure increased dose proportionally. No patient achieved disease control at 8 weeks, and all six patients developed progressive disease.

**Conclusions:**

Oleclumab was well tolerated in adult Japanese patients with advanced solid malignancies and no unexpected safety concerns were raised; oleclumab exposure increased with dose. Future studies on combination therapy with other agents are warranted.

**Supplementary Information:**

The online version contains supplementary material available at 10.1007/s10147-022-02242-5.

## Introduction

Cell death triggers release of adenosine triphosphate (ATP) from tumour cells into the extracellular space, leading to a pro-inflammatory response. To prevent an immune reaction stimulated by cell death, tissues express cluster of differentiation (CD)39 and CD73, which enzymatically convert ATP to adenosine monophosphate (AMP) and AMP to adenosine, respectively. Adenosine is an immunosuppressive metabolite [1] and CD73 overexpression is associated with poor prognosis in multiple cancer types [2–8]. Therefore, it is suggested that CD73-targeted immunotherapy and CD73 inhibition may reduce adenosine production, thus augmenting the host and/or immunotherapy response to tumours [9, 10].

Oleclumab is a human IgG1λ monoclonal antibody that potently and selectively inhibits the catalytic activity of CD73, and decreases CD73 expression through internalisation [11, 12]. Oleclumab is a potential treatment for multiple advanced tumour types, for which nonclinical evidence suggests a role of intra-tumoural adenosine in the tumour biology response. Oleclumab, alone and in combination with durvalumab, is currently under evaluation in an ongoing phase I clinical trial (NCT02503774) [13].

In this study, the primary objective was to assess the safety and tolerability of oleclumab in Japanese patients with advanced solid malignancies. The secondary objectives were to determine the pharmacokinetics, immunogenicity, biomarkers of oleclumab activity, and antitumour activity of oleclumab in this patient population.

## Patients and methods

### Study design

This phase I, single-centre, open-label study comprised two cohorts: Cohort 1 received oleclumab 1500 mg intravenously (IV) every 2 weeks (Q2W), and Cohort 2 received oleclumab 3000 mg IV Q2W. The study was conducted at the National Cancer Center Hospital (Japan) from November 2018 to June 2019. At least three and up to six evaluable Japanese patients with advanced solid malignancies were planned to be enrolled in each cohort.

Three evaluable patients were enrolled in each dose-level cohort; if no dose-limiting toxicity (DLT) was observed, safety assessments were conducted in all evaluable patients. If 1/3 patients experienced a DLT, the cohort would be expanded to six patients. If patients did not experience further DLTs in Cohort 1, they could proceed to the next cohort; in Cohort 2, the 3000 mg-dose would be considered the tolerated dose. Dose escalation would be terminated if a patient presented unacceptable toxicity or if ≥ 2 patients in a cohort experienced a DLT. How DLTs were defined is explained in Online Resource 1.

Patients received oleclumab IV on Day 1 and Day 15 in a 4-week cycle for as long as a clinical benefit was shown, unless the patient had progressive disease (PD) with either clinical deterioration and/or no further benefit from treatment, experienced unacceptable toxicity, or discontinued for any other reason.

This study was conducted in accordance with the Declaration of Helsinki and Council for International Organizations of Medical Sciences International Ethical Guidelines, applicable International Conference on Harmonisation Good Clinical Practice Guidelines, and applicable laws and regulations. All participants provided written informed consent prior to enrolment. The study protocol was approved by the Institutional Review Board, and the study was registered at ClinicalTrials.gov (NCT03736473).

### Patients

The inclusion criteria were as follows: male and female patients aged ≥ 20 years with histologically confirmed solid tumours refractory to standard therapy or for which no standard of care regimen exists; at least one lesion that was measurable using Response Evaluation Criteria in Solid Tumours (RECIST) v1.1 [14] (a previously irradiated lesion could be considered a target lesion if it was well defined, measurable per RECIST v1.1, and had clearly progressed); consented to provide archived tumour specimens or tumour biopsies for correlative biomarker studies; Eastern Cooperative Oncology Group (ECOG) performance status of 0 or 1; and adequate organ function.

The exclusion criteria were as follows: not having completed any anti-cancer treatments before enrolment; prior treatment with a CD73 antagonist; known allergy/hypersensitivity to the study drug; cardiac or peripheral vascular disease, uncontrolled massive ascites or pleural effusion, active or prior documented autoimmune disease, or untreated/unstable central nervous system metastatic disease; any concurrent chemotherapy, immunotherapy, or biological or hormonal therapy for cancer treatment; patients who were pregnant, lactating, or intended to become pregnant during their participation in the study; and considered unsuitable to participate by the investigator.

### Safety and tolerability

Safety endpoints included adverse events (AEs), serious AEs (SAEs), DLTs, vital signs, electrocardiogram (ECG) results, and laboratory parameters. AEs were recorded from the study start to the 90-day follow-up period after the last dose of oleclumab was administered. AEs were classified by Medical Dictionary for Regulatory Activities (MedDRA) version 22.0 System Organ Class (SOC) and Preferred Term (PT) and graded by the Common Terminology Criteria for Adverse Events (CTCAE) version 4.03. How SAEs were defined is included in Online Resource 1.

### Pharmacokinetic analysis

For pharmacokinetic analysis, serum concentrations of oleclumab were determined pre-dose, 10 min, and 2 h after oleclumab infusion on Day 1 (at first administration of oleclumab). For the second and third administrations of oleclumab (on Days 15 and 29, respectively), serum concentrations for oleclumab were determined pre-dose and 10 min after oleclumab infusion. Serum samples for the pharmacokinetic analysis were planned to be collected pre-dose and 10 min after oleclumab infusion on Day 57 and Day 113, but no patient continued oleclumab treatment up to Day 57. Serum concentrations for oleclumab were also determined at 30 and 90 days after the last dose.

### Immunogenicity

The development of detectable antidrug antibodies (ADAs) following treatment with oleclumab was evaluated. Serum samples for the assessment of ADAs were taken at the first and third administrations of oleclumab (on Days 1 and 29, respectively). Serum samples for ADAs were collected pre-dose on Day 1, and ADAs were also measured on Days 30 and 90 after the last dose.

### Biomarker analysis

Tumour tissue and blood samples were collected from all study participants for biomarker analysis, including CD73 expression. Adequate tumour tissue was defined as a formalin-fixed and paraffin-embedded (FFPE) tumour block or approximately 10 unstained slides, each 4–5 µm thick. An archival FFPE sample taken within 3 years prior to screening was preferred. Immunohistochemistry (IHC) of tissue biopsy specimens for CD73 and programmed death-ligand 1 (PD-L1) was performed. CD73 IHC was performed with the anti-CD73 antibody clone [EPR6115] (ab124725) and the Ventana Discover ULTRA autostainer (Ventana Medical Systems, Tucson, AZ, USA). PD-L1 IHC was performed with the anti-PDL1 antibody clone (SP263) and the Ventana Benchmark ULTRA autostainer. Following immunostaining, slides were scanned using an Aperio AT turbo or an Aperio XT scanner with a 20 × microscope objective. For blood samples, approximately 3.5 mL of whole blood was collected at each time point, from which > 0.5 mL of serum sample was obtained. The methods for ligand binding analysis of soluble CD73 are summarised in Online Resource 1.

### Antitumour activity

Antitumour activity was evaluated at 8 weeks based on the objective response rate (ORR) and disease control rate (DCR) assessed by RECIST v1.1. Clinical activity was evaluated by duration of response (DoR) and progression-free survival (PFS).

### Statistical methods

For sample size determination, three to six patients were planned to be enrolled in each cohort; those meeting eligibility criteria were enrolled; and the total number of patients was dependent on the result of the DLT assessment. The safety analysis set included all patients who received at least one dose of the study drug. The pharmacokinetic analysis set included all patients with measurable oleclumab concentrations and without important AEs or protocol deviations that may have affected the pharmacokinetics. The immunogenicity analysis set included all patients with baseline ADA results and at least one postbaseline ADA result. The efficacy analysis set included patients with tumour assessment data at baseline and who received at least one dose of the study drug. The biomarker analysis set included all patients who took part in the biomarker analysis.

Baseline demographic and clinical characteristics are summarised using mean ± standard deviation (SD) or median (range) for continuous variables and *n* (%) for categorical variables. Regarding pharmacokinetic data, serum concentrations of oleclumab were summarised by nominal sample time, cohort (e.g., dose level), and by visit and day. Pharmacokinetic parameters were summarised by cohort. Concentrations and pharmacokinetic parameters were summarised using descriptive statistics. Regarding immunogenicity analysis, results were listed by patient and analysed descriptively by summarising the number and percentage of patients who developed detectable anti-oleclumab antibodies. The immunogenicity titre was listed for samples that were positive for the presence of anti-oleclumab antibodies. Definitions for ORR, DCR, DoR, and PFS are included in Online Resource 1. The statistical analyses were performed using SAS version 9.3 (SAS Institute Inc., Cary, NC, USA).

## Results

### Patients

In total, six patients were enrolled in the study, and all six patients received the study treatment (three patients in Cohort 1 and three patients in Cohort 2). Two patients (one from each cohort) withdrew from the study prior to the 90-day follow-up visit, and four patients (two from each cohort) completed the study up to the 90-day follow-up visit. Protocol deviations were reported in four patients (three in Cohort 1 and one in Cohort 2). All six patients were included in the analysis sets (safety, pharmacokinetic, immunogenicity, efficacy, and biomarker analysis sets).

The baseline demographic and clinical characteristics of patients are summarised in Table 1. Overall, the median (range) age of the patients was 56.0 (51–77) years. All patients were Japanese and the majority (4/6 [66.7%]) were male. All six patients presented with different tumour types, and their disease stage, tumour stage, node stage, and metastasis stage also varied. All six patients discontinued treatment due to PD.

**Table 1:** Patient baseline demographic and clinical characteristics

	Oleclumab 1500 mg (*n* = 3)	Oleclumab 3000 mg (*n* = 3)	Total (*N* = 6)
Age, years			
Mean ± standard deviation	68.3 ± 9.6	52.3 ± 1.5	60.3 ± 10.7
Median (range)	70.0 (58–77)	52.0 (51–54)	56.0 (51–77)
Sex, *n* (%)			
Male	2 (66.7)	2 (66.7)	4 (66.7)
Female	1 (33.3)	1 (33.3)	2 (33.3)
ECOG performance status, *n* (%)			
0	2 (66.7)	2 (66.7)	4 (66.7)
1	1 (33.3)	1 (33.3)	2 (33.3)
Race Japanese, *n* (%)	3 (100.0)	3 (100.0)	6 (100.0)
Tumour type, *n* (%)			
Bile duct	0	1 (33.3)	1 (16.7)
Oesophagus	1 (33.3)	0	1 (16.7)
Orbital tumour	0	1 (33.3)	1 (16.7)
Ovarian	1 (33.3)	0	1 (16.7)
Pancreatic	1 (33.3)	0	1 (16.7)
Skin fibrosarcoma	0	1 (33.3)	1 (16.7)
UICC disease stage, *n* (%)			
III	1 (33.3)	1 (33.3)	2 (33.3)
IV	1 (33.3)	2 (66.7)	3 (50.0)
IVa	1 (33.3)	0	1 (16.7)

*ECOG* Eastern Cooperative Oncology Group; *UICC* Union for International Cancer Control

### Safety and tolerability

The median (range) total duration of treatment was 58 (57–58) days in Cohort 1 and 57 (57–57) days in Cohort 2. All patients in Cohorts 1 and 2 received four doses of the study treatment before discontinuation. No patient experienced a dose interruption/dose delay during either the DLT evaluation period or the rest of the study period.

A summary of AEs is shown in Table 2. All patients reported AEs during the study. One (16.7%) patient reported a Grade 3 AE (neutrophil count decreased) that was not considered related to the study treatment. No AEs with an outcome of death were reported, no SAEs were reported during the study, and no patients reported AEs or SAEs leading to oleclumab discontinuation or dose interruption. A summary of AEs by SOC and PT is shown in Online Resource 2. AEs with the highest incidence were decreased appetite, nausea, stomatitis, and rash, which were each reported in three (50.0%) patients.

**Table 2:** Summary of AEs

AE category	Oleclumab 1500 mg (*n* = 3)	Oleclumab 3000 mg (*n* = 3)	Total (*N* = 6)
Any AE	3 (100.0)	3 (100.0)	6 (100.0)
Any AE related to treatment	3 (100.0)	2 (66.7)	5 (83.3)
Any AE of CTCAE ≥ Grade 3	0	1 (33.3)	1 (16.7)
Any AE of CTCAE ≥ Grade 3, related to treatment	0	0	0
Any AE with an outcome of death	0	0	0
Any AE with an outcome of death, related to treatment	0	0	0
System Organ Class/Preferred Term			
Patients with AE of CTCAE Grade ≥ 3	0	1 (33.3)	1 (16.7)
Investigations	0	1 (33.3)	1 (16.7)
Neutrophil count decreased	0	1 (33.3)	1 (16.7)

All data are presented as *n* (%)

*AE* adverse event; *CTCAE* Common Terminology Criteria for Adverse Events

A summary of AEs possibly related to treatment by SOC and PT is shown in Table 3. Five (83.3%) patients reported AEs that were considered related to the study treatment. These AEs, which were each reported in one patient, included anaemia, decreased appetite, peripheral sensory neuropathy, orthostatic hypotension, epistaxis, nausea, stomatitis, nail disorder, rash, and elevated aspartate aminotransferase levels. No DLTs were reported. No clinically important changes in haematology parameters, clinical chemistry parameters, vital signs, or ECG parameters were observed (additional details provided in Online Resource 3).

**Table 3:** Number of patients with AEs possibly related to treatment by System Organ Class and Preferred Term

System Organ Class/Preferred Term	Oleclumab 1500 mg (*n* = 3)	Oleclumab 3000 mg (*n* = 3)	Total (*N* = 6)
Patients with any AE possibly related to treatment	3 (100.0)	2 (66.7)	5 (83.3)
Blood and lymphatic system disorders	0	1 (33.3)	1 (16.7)
Anaemia	0	1 (33.3)	1 (16.7)
Metabolism and nutrition disorders	0	1 (33.3)	1 (16.7)
Decreased appetite	0	1 (33.3)	1 (16.7)
Nervous system disorders	0	1 (33.3)	1 (16.7)
Peripheral sensory neuropathy	0	1 (33.3)	1 (16.7)
Vascular disorders	1 (33.3)	0	1 (16.7)
Orthostatic hypotension	1 (33.3)	0	1 (16.7)
Respiratory, thoracic, and mediastinal disorders	0	1 (33.3)	1 (16.7)
Epistaxis	0	1 (33.3)	1 (16.7)
Gastrointestinal disorders	0	2 (66.7)	2 (33.3)
Nausea	0	1 (33.3)	1 (16.7)
Stomatitis	0	1 (33.3)	1 (16.7)
Skin and subcutaneous tissue disorders	1 (33.3)	1 (33.3)	2 (33.3)
Nail disorder	0	1 (33.3)	1 (16.7)
Rash	1 (33.3)	0	1 (16.7)
Investigations	1 (33.3)	0	1 (16.7)
Aspartate aminotransferase increased	1 (33.3)	0	1 (16.7)

All data are presented as *n* (%)

Patients with multiple events in the same category were counted only once in that category

Patients with events in more than one category were counted once in each of those categories

*AE* adverse event

### Pharmacokinetics

**Fig. 1:**
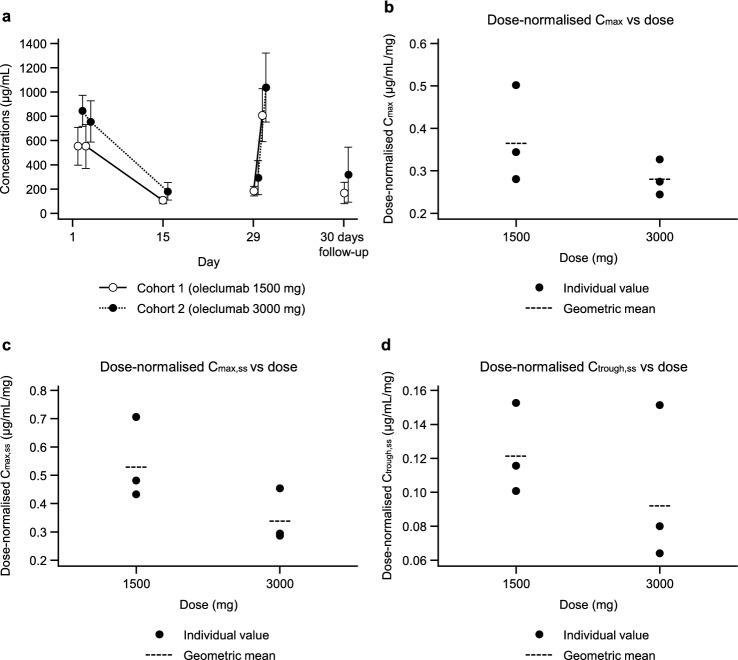
Arithmetic mean ± standard deviation serum concentrations (µg/mL) of oleclumab over time for Cohort 1 (1500 mg) and Cohort 2 (3000 mg) (log scale, *n* = 3 per dose level, pharmacokinetic analysis set) (**a**) and dose-normalised C_max_ (**b**), C_max,ss_ (**c**), and C_trough,ss_ (**d**) of oleclumab versus dose (pharmacokinetic analysis set). Abbreviations: C_max_, maximum serum concentration; C_max,ss_, maximum serum concentration at steady state; C_trough,ss_, trough serum concentration at steady state

The arithmetic mean (± SD) serum concentrations for oleclumab over time are shown in Fig. 1a. Pharmacokinetic parameters of oleclumab are summarised in Table 4. The exposure (maximum serum concentration [C_max_], area under the serum concentration–time curve from zero to the time of the last quantifiable concentration [AUC_0–t_], and trough serum concentration [C_trough_]) of oleclumab after a single dose showed a between-patient variability (coefficient of variation [CV]) of 14.8%–40.2%. The exposure (maximum serum concentration at steady state [C_max,ss_] and trough serum concentration at steady state [C_trough,ss_]) of oleclumab after multiple doses showed a between-patient variability (CV) of 21.7%–47.1%. After multiple (three times) dosing of oleclumab, the exposure of oleclumab increased from 1.2 to 1.5 and 1.6 to 1.7 in average accumulation ratios based on C_max_ and C_trough_, respectively.

**Table 4:** Summary of pharmacokinetic parameters of oleclumab 1500 mg and 3000 mg (pharmacokinetics analysis set)

Oleclumab	C_max_ (μg/mL)	t_max_ (day)	AUC_0–14 days_ (μg·day/mL)	AUC_0–t_ (μg·day/mL)	C_trough_ (μg/mL)	C_max,ss_ (μg/mL)	t_max,ss_ (day)	C_trough,ss_ (μg/mL)	R_AC_ (C_max_)	R_AC_ (C_trough_)
1500 mg										
*n*	3	3	2	3	3	3	3	3	3	3
Geometric mean	548.1		NC	3796.9	107.9	794.1		182.0		
CV (%)	30.3		NC	29.0	24.2	26.9		21.7		
Geometric SD	1.3		NC	1.3	1.3	1.3		1.2		
Arithmetic mean	564.5		NC	3897.4	110.0	812.3		184.8	1.5	1.7
SD	170.9		NC	1131.4	26.6	218.1		40.0	0.1	0.1
Median		0.1					0.1			
Min	422.1	0.1	3005.4	3114.7	87.3	652.3	0.1	151.4	1.4	1.7
Max	754.0	0.1	5174.7	5194.7	139.2	1060.8	0.1	229.1	1.6	1.7
3000 mg										
* n*	3	3	3	3	3	3	3	3	3	3
Geometric mean	841.7		5588.2	5610.2	173.2	1016.1		276.3		
CV (%)	14.8		28.8	28.6	40.2	27.3		47.1		
Geometric SD	1.2		1.3	1.3	1.5	1.3		1.6		
Arithmetic mean	847.8		5738.7	5759.8	182.5	1039.8		296.0	1.2	1.6
SD	125.5		1652.4	1647.4	73.3	284.1		139.4	0.2	0.1
Median		0.1					0.1			
Min	735.1	0.1	4373.3	4384.6	120.1	864.5	0.1	193.1	1.1	1.5
Max	983.0	0.1	7575.7	7585.6	263.3	1367.6	0.1	454.7	1.4	1.7

*AUC* area under the concentration–time curve; *AUC*_*0–14 days*_ AUC from 0 to 14 days after initiation of oleclumab infusion; *AUC*_*0–t*_ AUC from zero to the time of the last quantifiable concentration; *C*_*max*_ maximum serum concentration;* C*_*max,ss*_ C_max_ at steady state; *C*_*trough*_ trough serum concentration; *C*_*trough,ss*_ C_trough_ at steady state; *CV* coefficient of variation; *NC* not calculated; *R*_*AC*_ accumulation ratio; *SD* standard deviation; *t*_*max*_ time to maximum plasma concentration; *t*_*max,ss*_ t_max_ at steady state

The dose proportionality of oleclumab exposure was assessed by numerical and visual inspection because the study drug was administered at only two dose levels. The ratios of geometric means for the pharmacokinetic parameters (3000 mg/1500 mg) were 1.54 for C_max_, 1.28 for C_max,ss_, and 1.52 for C_trough,ss_ against a twofold increase in dose from 1500 to 3000 mg. Graphical representations of dose-normalised values of each parameter (C_max_, C_max,ss_, and C_trough,ss_) of oleclumab versus the dose are presented in Fig. 1b–d. Data suggested that the exposures (C_max_, C_max,ss_, and C_trough,ss_) of oleclumab after both single and multiple doses increased in a less than dose-proportional manner.

### Immunogenicity

Ten and 11 samples collected from each of the three patients in Cohorts 1 and 2, respectively, were tested for the presence of ADAs against oleclumab. All the samples were determined to be negative for ADAs against oleclumab.

### Biomarker analysis

**Fig. 2:**
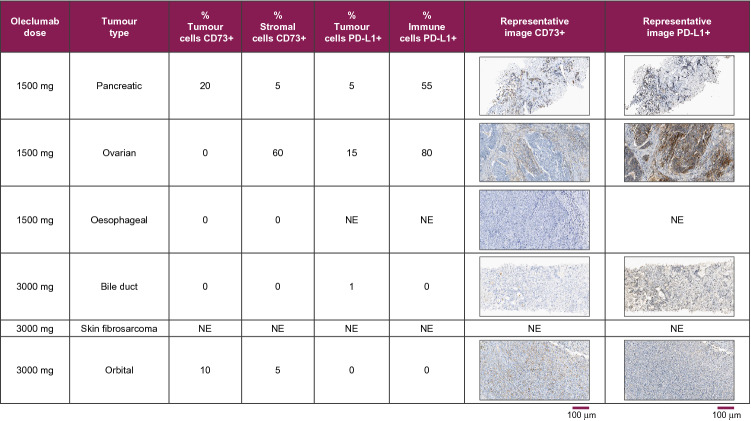
Summary of CD73 and PD-L1 expression from archival tumour samples. PD-L1 immunohistochemistry (IHC) was performed using an anti-PD-L1 antibody clone (SP263), and CD73 IHC was performed using an anti-CD73 antibody clone (EPR6115). Abbreviations: CD73, cluster of differentiation 73; NE, not evaluable; PD-L1, programmed death-ligand 1

IHC of tissue biopsy specimens for CD73 and PD-L1 was performed, and the results are summarised in Fig. 2. No association was observed between these parameters and clinical activity, as there was no therapeutic activity observed and the cohort size was small. Free soluble CD73 in the blood of all patients decreased 15 days after dosing (data not shown).

### Antitumour activity

No patient achieved disease control at 8 weeks and all six patients developed PD. The best percent change from baseline in target lesion size is shown in Online Resource 4. No patients experienced a reduction in target lesion size from baseline in either cohort. The best percentage change in an individual patient was an increase of less than 20% (15.4%), but the best objective response of the patient was judged as PD because a new lesion was found. The best percentage change in target lesion size overall was a median (range) increase of 23.7% (15.4%–64.7%) in Cohort 1 and a median (range) increase of 34.1% (34.1%–41.0%) in Cohort 2.

## Discussion

This phase I, single-centre, open-label study assessed the safety of oleclumab in Japanese patients with advanced solid malignancies who were not responding to standard treatments or for whom no standard of care regimen existed. From the results of the phase I study D6070C00001, the recommended dose regimen for oleclumab was 3000 mg Q2W for the first four doses followed by 3000 mg Q4W. Considering there were no studies on treating Japanese patients with oleclumab, the starting dose of oleclumab was 1500 mg Q2W, which was − 1 dose level from 3000 mg Q2W and is the current dose used in the global study (NCT04068610, NCT03611556). Six patients were enrolled in the present study, and all of them discontinued treatment due to PD. In line with previous data, oleclumab was tolerable in this patient population and no new safety concerns were raised.

In terms of safety, no deaths, SAEs, or AEs leading to discontinuation were reported; all patients reported AEs, among which the most frequent were decreased appetite, nausea, stomatitis, and rash; no Grade 4 AEs or DLTs were reported, and no patient discontinued due to an AE related to oleclumab. Oleclumab monotherapy demonstrated a manageable safety profile in patients with advanced solid malignancies. Overall, clinical laboratory and other safety assessments were in line with the reported safety profile of oleclumab when administered alone [13]. A previous phase I study showed that oleclumab monotherapy had a tolerable safety profile; no DLTs were observed, and the incidence of Grade 3–4 treatment-related AEs was 7.1% [13]. However, oleclumab in combination with durvalumab or osimertinib was shown to increase the incidence of AEs in previous studies: in a previous phase I study, no DLTs were reported when administered in combination with durvalumab, but the incidence of Grade 3–4 treatment-related AEs was 20.8% [13]; in another phase II study, the incidence of Grade ≥ 3 treatment-emergent AEs was 40.7% when administered in combination with durvalumab [15]; in a previous phase Ib/II study, the incidence of Grade 3–4 treatment-emergent AEs was 38.1% when administered in combination with osimertinib [16]; and in another phase I/II study, five out of six patients experienced Grade ≥ 3 neutropenia outside of the DLT period when administered in combination with durvalumab and chemotherapy [17]. In these studies, it was concluded that the combination treatment was tolerable [13, 15–17]. However, direct comparisons with the present study are difficult because of differences in study design and the types of cancers included in the studies.

The pharmacokinetic results showed that there was low to modest variability for oleclumab exposure among patients. There were no observed marked differences in the variability between doses (1500 mg versus 3000 mg) or between single and multiple doses. From numerical and visual inspection, exposure to oleclumab after both single and multiple doses increased in accordance with the increase in dose, however the increase was less than dose proportional.

Regarding immunogenicity, all the analysed samples tested negative for ADAs. In the biomarker analysis, no association was observed for the level of CD73 and PD-L1 expression in tissue samples or for clinical response, as no therapeutic activity was observed, and the cohort size was small.

Regarding the antitumour effect of oleclumab, all enrolled patients developed PD and no patient achieved disease control at 8 weeks. No patient showed a decrease in target lesion size. Although oleclumab monotherapy did not show positive clinical responses in any patients, it should be noted that the patient number was too small, and the follow-up duration was too short in this study to draw any definitive conclusions. In addition, in this short follow-up period, tumour pseudoprogression cannot be totally excluded [18, 19]. Previous studies showed promising antitumour activity of oleclumab when administered in combination with other drugs. In a previous phase I study, objective response was observed in four non-small-cell lung cancer patients with epidermal growth factor receptor mutation, and the DoR range was 5.6 to 15.7 + months when oleclumab was administered in combination with durvalumab [13]. In another phase II study of oleclumab administered in combination with durvalumab, ORR was 30.0% in patients with Stage III non-small-cell lung cancer after concurrent chemoradiation therapy [15]. In a previous phase Ib/II study of oleclumab administered in combination with osimertinib, ORR was 19% and median PFS was 11 months [16], and in another phase I/II study, four out of six patients with locally advanced unresectable or metastatic triple-negative breast cancer who were treated with oleclumab administered in combination with durvalumab and chemotherapy had a clinical benefit [17].

Considering the mode of action of oleclumab, the combination of oleclumab with other immuno-oncology agents (e.g., antibodies against programmed death-1/PD-L1) might exert meaningful clinical effects; thus, identification of the patient population most likely to derive benefit is clinically desirable. Multiple preclinical studies have shown that the combination of PD-L1 antibody with CD73 antibody demonstrated better efficacy compared with each monotherapy [10, 20–22]. Interestingly, the combination of PD-L1 and CD73 antibody enhanced gene signatures associated with T cell-mediated cytotoxicity, inflammation, leukocyte infiltration, interferon-γ response, and type I interferon response. Oleclumab is likely to be able to effectively combine with other immunotherapies (e.g., PD-1/PD-L1 signalling pathway) because of complementarity of the mechanisms of action. Additionally, CD73 upregulation after PD-1 therapy has been reported in several studies [10, 23, 24], and treatment with a CD73 blocking antibody has a potential to overcome PD-1 therapy-induced immune escape status. Therefore, we find it necessary to understand PD-L1 expression in the context of treatment and response, and the combination of PD-L1 antibody with CD73 antibody may be the preferable approach to beat the current limitation due to the complicated immune suppressive pathways in the tumour microenvironment. Treatment of tumour cells with paclitaxel, carboplatin, doxorubicin, or gemcitabine resulted in induction of PD-L1 and CD73 expression, providing a strong rationale for combining chemotherapy with anti-PD-L1 and anti-CD73 antibodies [25].

In preclinical in vitro and in vivo studies, gemcitabine effectively inhibited tumour growth by inducing immunogenic cell death in pancreatic cancer and increased ATP and HMGB1 release [26]. In another preclinical study on multiple chemotherapeutic agents and multiple triple-negative breast cancer cell lines, chemotherapy induced transcriptional activation of PD-L1, CD47, and CD73 expression. The cell surface expression of PD-L1 and/or CD73 on triple-negative breast cancer cells enables anergy or apoptosis of effector T cells with a concomitant increase in regulatory T cells in the tumour microenvironment, thus impairing adaptive antitumour immunity [25]. These preclinical studies suggest that chemotherapy may increase extracellular ATP and increase CD73 and PD-L1 expression. This supports the combination of immunotherapy targeting CD73 and/or PD-L1 with chemotherapy to improve efficacy.

Another in vitro study showed that targeted blockade of CD73 may enhance the therapeutic activity of anti-PD-1 and anti-CTLA-4 monoclonal antibodies and may potentiate the activity of this combination [20]. In addition, treatment of tumour-bearing mice with oleclumab combined with an anti-PD-1 antibody resulted in greater antitumour activity than either antibody used as monotherapy [12]. These observations suggest that an inhibitor of adenosine production in combination with a checkpoint inhibitor may generate superior antitumour activity when compared with either agent as monotherapy, which may yield more complete and more durable antitumour activity.

This study has some limitations, including those inherent to phase I studies, such as the small sample size and the single-centre study design. Moreover, four patients failed to adhere to the study protocol.

## Conclusions

Oleclumab (1500 mg and 3000 mg) was well tolerated when administered IV Q2W in this study population of adult Japanese patients with advanced solid malignancies, and no unexpected safety concerns were raised. Oleclumab exposure increased in accordance with the increase in dose after both single and multiple doses, but this was less than proportional to the dose; all samples from all patients were determined to be negative for the presence of ADAs. All patients showed disease progression at the first on-treatment evaluation (~ 8 weeks). Current findings are expected to form the basis for decisions of future studies in Japan. Considering that oleclumab was well tolerated, showed a favourable safety profile, and considering its mode of action, future studies on combination therapy with other agents may be worthwhile.

## Supplementary Information

Below is the link to the electronic supplementary material.

Supplementary file1 (PDF 246 KB)

## Data Availability

Data underlying the findings described in this manuscript may be obtained in accordance with AstraZeneca’s data sharing policy described at http://astrazenecagrouptrials.pharmacm.com/ST/Submission/Disclosure.
