# Supplementary material for: Safety, tolerability, pharmacokinetics, and antitumour activity of oleclumab in Japanese patients with advanced solid malignancies: a phase I, open-label study
Source: Int J Clin Oncol. 2022 Nov 7;27(12):1795–804. doi: 10.1007/s10147-022-02242-5 (PMC9700577; doi:10.1007/s10147-022-02242-5)
Supplement: Supplementary file 1 — Supplementary file1 (PDF 246 KB) [file 10147_2022_2242_MOESM1_ESM.pdf]

International Journal of Clinical Oncology
Electronic Supplementary Material

Safety, tolerability, pharmacokinetics, and antitumour activity of oleclumab in Japanese patients with advanced solid malignancies: a phase I, open-label study

Shunsuke Kondo,[1] Satoru Iwasa,[1] Takafumi Koyama,[1] Tomoko Fujita,[2] Ko Sugibayashi,[3,4] Kosho Murayama,[2] Noboru Yamamoto[1]

1. National Cancer Center Hospital, 5-1-1, Tsukiji, Chuo-ku, Tokyo 104-0045, Japan
2. AstraZeneca K.K., 3-1, Ofuka-cho, Kita-ku, Osaka 530-0011, Japan
3. AstraZeneca K.K., 3-1-1, Shibaura, Minato-ku, Tokyo 108-0023, Japan
4. Present Address: AstraZeneca, 136 Hills Road, Cambridge, CB2 8PA, UK

Corresponding author

Shunsuke Kondo, MD

National Cancer Center Hospital, 5-1-1, Tsukiji, Chuo-ku, Tokyo 104-0045, Japan

Tel: +81-3-3542-2511

Fax: +81-3-3542-3815

E-mail: shkondo@ncc.go.jp

Online Resource 1

Definition of dose-limiting toxicity (DLT)

DLT was defined as any of the events listed below that occurred during the DLT-evaluation period (defined as the time from receiving the first dose of oleclumab until assessments prior the planned administration of the third dose on Day 29). Toxicity that was clearly and directly related to the primary disease or to another aetiology was excluded from this definition. The following were considered DLTs:

- Grade 4 immune-mediated adverse event (AE)
- Grade ≥ 3 colitis
- Grade ≥ 3 nausea, vomiting, or diarrhoea that does not resolve to Grade ≤ 2 within 3 days of the initiation of maximal supportive care
- Grade ≥ 3 pneumonitis or interstitial lung disease
- Grade 4 anaemia or anaemia requiring red cell transfusion
- Grade 4 thrombocytopenia or neutropenia that is present for more than 4 days
- Grade 3 thrombocytopenia with bleeding or thrombocytopenia requiring platelet transfusion
- Febrile neutropenia
- Isolated liver transaminase elevation ≥ 5 × but ≤ 8 × upper limit of normal (ULN) or isolated total bilirubin ≥ 3 × but ≤ 5 × ULN that does not downgrade to Grade ≤ 2 within 14 days after onset with optimal medical management, including systemic corticosteroids
- Isolated liver transaminase elevation > 8 × ULN or isolated total bilirubin > 5 × ULN regardless of duration
- Increase in aspartate aminotransferase or alanine aminotransferase > 3 × ULN and concurrent increase in total bilirubin > 2 × ULN (Hy's Law) without evidence of cholestasis or alternative explanations (e.g., viral hepatitis, disease progression in the liver)
- Any other toxicity that is greater than that at baseline, is clinically significant, and/or unacceptable, and is judged to be a DLT by the Safety Review Committee.

Definition of serious AE (SAE)

An SAE was defined as an AE occurring during any study phase (i.e., run-in, treatment, washout, or follow-up period), that fulfilled one or more of the following criteria:

- Results in death
- Is immediately life-threatening
- Requires hospitalisation or prolongation of existing hospitalisation
- Results in persistent or significant disability or incapacity
- Results in a congenital abnormality or birth defect
- Is an important medical event that may jeopardise the subject or may require medical treatment to prevent one of the outcomes listed above.

Ligand binding analysis of soluble CD73

The procedure comprised a stepwise format in which wash steps followed each incubation. A human anti-CD73 monoclonal antibody (mAb) coated to a microtiter plate was used to capture free soluble CD73 in calibration samples, quality controls, and serum samples. Any bound soluble CD73 was then detected using a horseradish peroxidase labelled anti-CD73 mAb (non-competing with capture mAb). TMB (3,3′,5,5′-tetramethylbenzidine) was used as the substrate for the colorimetric reaction to quantitatively measure the binding complex; chromogenic colour development was directly related to levels of soluble CD73 in samples. Once the procedure had been stopped with acid, the plate was read on a spectrophotometer at 450 nm, and data were analysed with SoftMax® Pro (SMP), version 5.4. Data were fitted using a 5-parameter logistic function with no weighting factor, and concentrations of soluble CD73 in samples were measured by interpolation from the standard curve. The quantitative range of this assay was 0.25 ng/mL (lower limit of quantitation) to 15 ng/mL (upper limit of quantitation).

Outcome definitions

Regarding the efficacy analysis, the objective response rate was defined as the proportion of patients with confirmed complete response (CR) or confirmed partial response (PR); disease control rate was defined as the proportion of patients with CR, PR, or stable disease for ≥8 weeks; duration of response was defined as the duration from the first documentation of objective response to the first documented disease progression or death due to any cause, whichever occurred first; and progression-free survival was measured from the start of treatment until the first documentation of disease progression or death due to any cause, whichever occurred first.

Online Resource 2 AEs by SOC and PT

System Organ Class / Preferred Term	Oleclumab 1500 mg (n = 3)	Oleclumab 3000 mg (n = 3)	Total (N = 6)
Patients with any AE	3 (100.0)	3 (100.0)	6 (100.0)
Infections and infestations	0	2 (66.7)	2 (33.3)
Conjunctivitis	0	1 (33.3)	1 (16.7)
Nasopharyngitis	0	1 (33.3)	1 (16.7)
Blood and lymphatic system disorders	0	1 (33.3)	1 (16.7)
Anaemia	0	1 (33.3)	1 (16.7)
Metabolism and nutrition disorders	1 (33.3)	2 (66.7)	3 (50.0)
Decreased appetite	1 (33.3)	2 (66.7)	3 (50.0)
Nervous system disorders	0	1 (33.3)	1 (16.7)
Peripheral sensory neuropathy	0	1 (33.3)	1 (16.7)
Vascular disorders	2 (66.7)	0	2 (33.3)
Hot flush	1 (33.3)	0	1 (16.7)
Orthostatic hypotension	1 (33.3)	0	1 (16.7)
Respiratory, thoracic, and mediastinal disorders	0	1 (33.3)	1 (16.7)
Epistaxis	0	1 (33.3)	1 (16.7)
Gastrointestinal disorders	3 (100.0)	3 (100.0)	6 (100.0)
Constipation	2 (66.7)	0	2 (33.3)
Dental caries	1 (33.3)	0	1 (16.7)
Nausea	1 (33.3)	2 (66.7)	3 (50.0)
Stomatitis	0	3 (100.0)	3 (50.0)
Vomiting	1 (33.3)	1 (33.3)	2 (33.3)
Skin and subcutaneous tissue disorders	2 (66.7)	1 (33.3)	3 (50.0)
Alopecia	1 (33.3)	0	1 (16.7)

Nail disorder	0	1 (33.3)	1 (16.7)
Rash	2 (66.7)	1 (33.3)	3 (50.0)
General disorders and administration site conditions	1 (33.3)	1 (33.3)	2 (33.3)
Pyrexia	1 (33.3)	1 (33.3)	2 (33.3)
Investigations	1 (33.3)	1 (33.3)	2 (33.3)
Alanine aminotransferase increased	1 (33.3)	1 (33.3)	2 (33.3)
Amylase increased	1 (33.3)	0	1 (16.7)
Aspartate aminotransferase increased	1 (33.3)	1 (33.3)	2 (33.3)
Neutrophil count decreased	1 (33.3)	1 (33.3)	2 (33.3)

All data are presented as n (%).

Abbreviations: AE, adverse event; PT, Preferred Term; SOC, System Organ Class.

Online Resource 3 Additional details of the safety results

One patient presented with a Grade 2 adverse event (AE) of neutrophil count decreased that was not considered related to the study treatment and was reported as recovered/resolved. One patient presented with a Grade 3 AE of neutrophil count decreased that was not considered related to the study treatment and was reported as not recovered/not resolved. One patient presented with a Grade 1 AE of anaemia that was considered related to the study treatment and reported as recovered/resolved.

No clinically important changes in clinical chemistry parameters were observed, except for a Grade change of ≥ 2 in gamma-glutamyl transferase reported in a patient in the oleclumab 3000 mg cohort (Cohort 2). One patient presented with a Grade 1 AE of elevated aspartate aminotransferase (AST) that was considered related to the study treatment and reported as recovered/resolved. This patient subsequently presented with Grade 1 elevated levels of alanine aminotransferase (ALT), amylase, and AST, which were not considered related to the study treatment and reported as not recovered/not resolved. One patient presented with Grade 1 elevated ALT and AST, which were not considered related to the study treatment and were reported as not recovered/not resolved.

No clinically important changes in vital signs were observed. One patient presented with a Grade 1 AE of orthostatic hypotension that was not considered related to the study treatment and was reported as recovered/resolved. One patient in Cohort 1 shifted from a baseline Eastern Cooperative Oncology Group performance status of 0 to 1 during the study. No clinically important changes in electrocardiogram parameters were observed.

Online Resource 4 Best percentage change from baseline in target lesion size (efficacy analysis set)

	**Oleclumab 1500 mg (n = 3)**	**Oleclumab 3000 mg (n = 3)**
Mean ± standard deviation	34.6 ± 26.4	36.4 ± 4.0
Median (range)	23.7 (15.4–64.7)	34.1 (34.1–41.0)
Proportion with reduction	0.0	0.0
Proportion with reduction > 10%	0.0	0.0
